# Case report: unilateral absence of the left pulmonary artery with left gastric artery collateral supply and hematologic disorder

**DOI:** 10.1093/omcr/omae209

**Published:** 2025-03-20

**Authors:** Naomie Condé, Mathew Carias, Josephine Pressacco

**Affiliations:** Department of Medicine, McGill University, 845 Sherbrooke W, Montreal (QC), H3A 0G4, Canada; Department of Radiology, McGill University Health Center, 1001 Bd Décarie, Montreal (QC), H4A 3J1, Canada; Department of Radiology, McGill University Health Center, 1001 Bd Décarie, Montreal (QC), H4A 3J1, Canada

**Keywords:** radiology, thoracic imaging, vasculature

## Abstract

Unilateral absence of the pulmonary artery is a congenital disorder resulting from malformation of the sixth aortic arch during embryogenesis. This case report presents a unique instance of unilateral absence of the pulmonary artery, incidentally discovered in an individual with multiple myeloma. Despite a history of recurrent childhood pulmonary infections, the patient remained asymptomatic until presenting with symptoms of myeloma. Imaging revealed a right-sided aortic arch, absent left pulmonary artery, and left lung atrophy with atelectasis. Among various collateral arteries, a notable finding in this case is the left gastric artery supplying the left lower lobe.

## Introduction

Unilateral absence of a pulmonary artery (UAPA), also known as unilateral pulmonary artery agenesis (UPAA), is a rare congenital condition with an estimated prevalence of 1 in 200 000 [1]. It is often associated with cardiac abnormalities and results from a malformation of the sixth aortic arch on the affected side during embryogenesis [2]. Due to the absence of a pulmonary artery, collateral arteries supply the affected lung. This condition typically presents with dyspnea and is often diagnosed in adolescence. However, some individuals may remain asymptomatic or undiagnosed until later in life. UAPA can present with recurrent infections or hemoptysis.

The objective of this case report is to present a unique case characterized by extensive collateral vascular supply, which has not been previously reported in the literature, along with a history of hematologic disorder, myeloma. The UAPA was an incidental finding, as the patient did not present with typical symptoms.

## Case presentation

The patient’s medical history includes hypertension and recurrent bronchopneumonia during childhood. They have remained asymptomatic until presenting with symptoms of multiple myeloma.

They presented to the emergency department with six months of progressive fatigue, a 20-pound weight loss, and left leg swelling extending from the thigh downward, with visibly dilated veins on the inner thigh. Deep vein thrombosis was ruled out with a Doppler ultrasound, and a Computed Tomography (CT) scan of the chest, abdomen, and pelvis were ordered to rule out malignancy.

**Figure 1:**
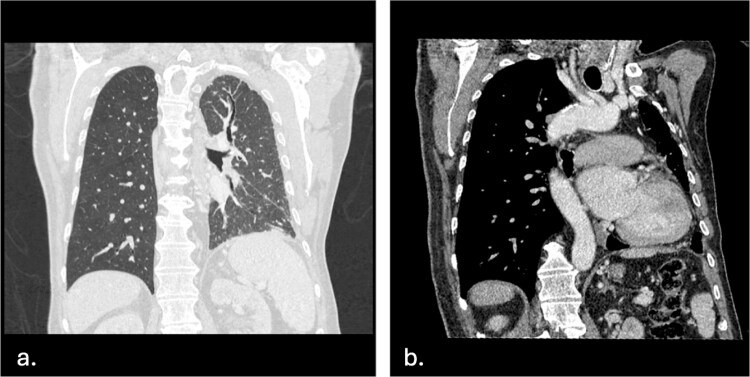
a. Left lung atelectasis and left-sided fibrothorax—Single coronal image in lung window demonstrates a reduced left lung size, with atelectasis suggestive and fibrothorax, likely due to recurrent pulmonary infections and UAPA. b. Right-sided aortic arch with mirror image—This coronal image shows a right-sided aortic arch with mirror image of the arch vessels.

The CT scan revealed multiple abnormalities including: calcified left pleural plaques, decreased left lung volume with diffuse reticulations, areas of subsegmental atelectasis, and left-sided fibrothorax (Fig. 1a). There was an incidental finding of UAPA with right-sided aortic arch and a mirror image of the arch vessels (Fig. 1b).

**Figure 2:**
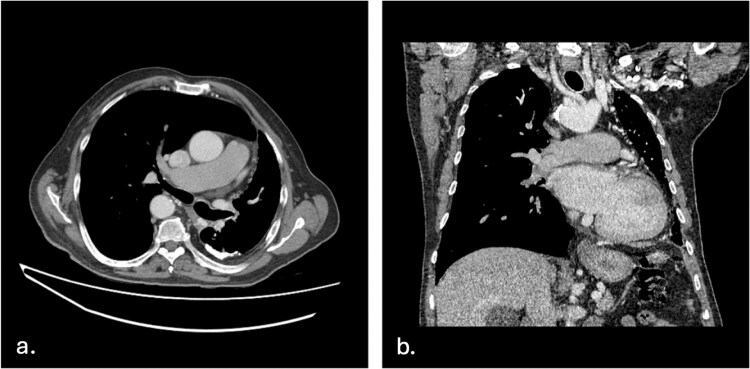
**Absent left pulmonary artery** a. axial plane—Axial image showing a dilated pulmonary trunk and right pulmonary artery (measuring up to 3.2 cm), suggestive of mild pulmonary hypertension, and an absent left pulmonary artery along with a visibly atrophied left lung and hypertrophied right lung. b. Coronal plane—Coronal image of the absent left pulmonary artery.

**Figure 3:**
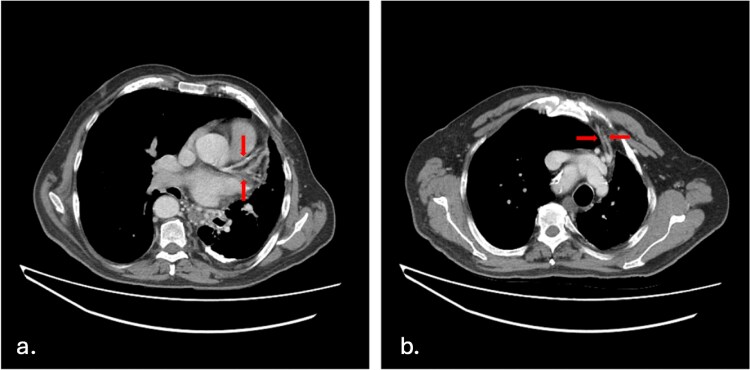
a. **Aortic collateral arteries**—Axial image showing significant aortic collaterals providing blood supply to the left upper lobe. b. Internal mammary collateral arteries—Axial image shows collateral arteries branching off the left internal mammary artery to supply the left lung.

**Figure 4:**
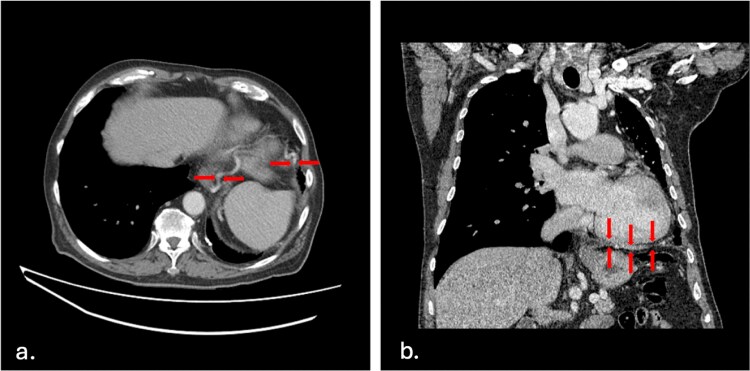
**Left gastric artery collateral supply** a. axial plane—This cut shows a tortuous left gastric artery superiorly traversing the esophageal hiatus supplying the left lower lobe. b. Coronal plane—Coronal image showing left gastric artery as it runs above the diaphragm, at the lower border of the left lung, after traversing the esophageal hiatus.

As a result of the UAPA, there was compensatory dilatation of the pulmonary trunk, measuring up to 3.2 cm, and the right central pulmonary artery (Fig. 2). Significant bronchial and aortic collaterals were present (Fig. 3a), as well as collateral supply from the left internal mammary artery, feeding the left upper lobe (Fig. 3b) and the left gastric artery supplying the left lower lobe (Fig. 4), a finding not previously described in the literature.

In this patient’s case, the unilateral absence of the pulmonary artery was an incidental finding as the patient did not present with any respiratory or cardiac symptoms. They were further evaluated for malignancy and laboratory investigations revealed microcytic anemia and myeloma following a bone marrow biopsy performed by the hematology team.

## Discussion

UAPA is a rare congenital condition. Sixty percent (60%) of cases are associated with other congenital heart defects, tetralogy of Fallot being the most common [3]. This case is particularly notable as it appears to be the first documented case of a patient presenting with both a unilateral absence of the left pulmonary artery and a right-sided aortic arch, alongside distinctive collateral vessels and a diagnosis of multiple myeloma.

Recurrent respiratory infections occur in 35.4% of cases involving UAPA [4], as demonstrated in the case presented. Although childhood medical records are unavailable, the patient reported a prolonged episode of bronchopneumonia that significantly affected the left lung. Additionally, radiographic imaging reveals areas of fibrothorax and atelectasis, consistent with this history.

The patient’s left leg edema may be related to pulmonary hypertension associated with UAPA, which can cause venous distention and peripheral edema, as was observed on a lower extremity Doppler ultrasound.

Regarding the vessels, it is not uncommon to observe a right-sided aortic arch in patients with UAPA [5]. Furthermore, previous studies showed coronary collaterals, as well as collaterals from intercostal and paravertebral arteries, and the right internal mammary artery in cases of right pulmonary artery agenesis. [6] In this case, collaterals from the left internal mammary artery, as well as aortic and bronchial branches, supply the upper lobe, suggesting adaptational angiogenesis to perfuse the left lung.

What is unique about this case is the left gastric artery feeding the left lower lobe, a finding not previously documented in the context of UAPA. Clinically, the left gastric artery is responsible for 85% of upper gastrointestinal hemorrhage [7]. Interestingly, a case discussing the left gastric artery supplying segments of the lung was reported in two patients presenting with life-threatening massive hemoptysis and subsequent embolization [8]. Some UAPA cases present with hemoptysis due to excessive collateral circulation. In a study of UAPA patients, hemoptysis was present in 41.5% of cases and was the most common symptom in patients with isolated UAPA in adulthood [4].

There has been no evidence suggesting an association between congenital vascular defects or UAPA and myeloma. However, studies indicate that multiple lines of therapy for multiple myeloma increase susceptibility to viral respiratory tract infections [9]. Moreover, corticosteroids, commonly used in multiple myeloma treatment, compromise immune function. Combined with the predisposition to pulmonary infections from UAPA, these factors place the patient at significant risk for respiratory complications.

Treatment options for UAPA are limited. Procedural options are mostly performed during childhood, including revascularization (systemic-pulmonary shunt), lobectomy, and embolization of aorto-pulmonary collateral arteries to prevent hemorrhage. [10] Medical management is used to address the complications of UAPA such as pulmonary hypertension and subsequent heart failure. For this patient, a transesophageal echocardiogram has been scheduled to evaluate for additional congenital anomalies and complications of UAPA.

## Conclusion

While the coexistence of UAPA and multiple myeloma in this patient appears coincidental, further research may be warranted to explore any potential links between congenital vascular anomalies and hematologic malignancies.

The presence of UAPA should prompt careful assessment for associated congenital anomalies and consideration of potential complications, such as pulmonary hypertension and recurrent pulmonary infections. Additionally, the discovery of unusual collateral circulation patterns, like that observed in this patient with the left gastric feeding the left lower lobe, pose a higher risk for hemorrhage, which may have implications for surgical planning or interventional procedures.

Clinicians should also be aware that UAPA may present asymptomatically or with non-specific symptoms, making its diagnosis challenging. Early identification and monitoring of patients with UAPA can help manage potential complications and improve outcomes.
